# A Fuzzy Clustering Approach to Identify Pedestrians’ Traffic Behavior Patterns

**DOI:** 10.34172/jrhs.2023.127

**Published:** 2023-09-29

**Authors:** Parisa Saeipour, Parvin Sarbakhsh, Saman Salemi, Fatemeh Bakhtari Aghdam

**Affiliations:** ^1^Department of Statistics and Epidemiology, Faculty of Health, Tabriz University of Medical Sciences, Tabriz, Iran; ^2^Department of Medicine, Islamic Azad University Tehran Medical Sciences, Tehran, Iran; ^3^Road Traffic Injury Research Center, Tabriz University of Medical Sciences, Tabriz, Iran

**Keywords:** Machine learning, Fuzzy, Cluster analyses, Behavior, Traffic crashes

## Abstract

**Background:** Pattern recognition of pedestrians’ traffic behavior can enhance the management efficiency of interested groups by targeting access to them and facilitating planning via more specific surveys. This study aimed to evaluate the pedestrians’ traffic behavior pattern by fuzzy clustering algorithm and assess the factors related to higher-risk traffic behavior of pedestrians.

**Study Design:** This study is a secondary methodological study based on the data from a cross-sectional study.

**Methods:** The fuzzy c-means (FCM), as a machine learning clustering method, was conducted to identify the pattern of traffic behaviors by collecting data from 600 pedestrians in Urmia, Iran via "the Pedestrian Behavior Questionnaire" (PBQ) and using 5 domains of PBQ. Multiple logistic regression was fitted to identify risk factors of traffic behaviors.

**Results:** Results revealed two clusters consisting of lower-risk and higher-risk behaviors. The majority of pedestrians (64.33%) were in the lower-risk cluster. Subjects≤33 years old (Odds ratio [OR]=1.92, *P*<0.001), subjects with≤6 years of education (OR=1.74, *P*=0.010), males (OR=1.90, *P*=0.001), unmarried pedestrians (OR=3.61, *P*=0.007), and users of public transportation (OR=2.01, *P*=0.002) were more likely to have higher-risk traffic behavior.

**Conclusion:** We identified traffic behavior patterns of Urmia pedestrians with lower-risk and higher-risk behaviors via FCM. The findings from this study would be helpful for policymakers to promote safety measures and train pedestrians.

## Background

 According to the reports of the World Health Organization (WHO), traffic accidents are one of the most common causes of injury and death.^1-3^ Existing research has found that a wide range of factors may contribute to the occurrence and severity of pedestrian crashes, including behavioral, roadway, and environmental factors.^4,5^

 Traffic behavior of pedestrians has always been one of the most important issues affecting traffic accident mortality. The risky behavior of a pedestrian can directly increase the chances of a traffic crash. Pattern recognition of traffic behaviors of pedestrians and identifying homogeneous groups have been the research subjects in several prior studies, and the traffic behavior analysis has always attracted considerable interest from transport authorities since understanding pedestrians in terms of their traffic behavior patterns can help the planning and implementation of better services. In addition, identifying such patterns of traffic behavior can enhance management efficiency via more targeted access to groups of interest and facilitate planning through more specific surveys.

 Furthermore, identifying clusters of pedestrians where each cluster presents a similar traffic behavior pattern can help to perform a more detailed evaluation through complementary analysis such as logistic regression to identify factors affecting their behavior and predict the probability of a pedestrian exhibiting more risky traffic behavior.^6^

 On the other hand, pedestrians’ traffic behavior data is too heterogeneous, and this heterogeneity makes it difficult to identify certain patterns and evaluate factors that significantly impact these behavioral patterns. Such heterogeneity may lead to biased estimation of parameters and potentially incorrect conclusions.^7,8^ Therefore, by building and applying more efficient models for pattern recognition in traffic data, researchers try to overcome the heterogeneity in these data to some extent.^9-11^ Furthermore, data segmentation techniques such as cluster analysis can help to explore hidden patterns in complex data sets^12^ such as pedestrians’ behavior data by reducing the heterogeneity of data.^13,14^

 There are two kinds of clustering methods: hard and soft clustering. Hard clustering methods similar to the k-means algorithm are suitable for limited clustering tasks in which each data point belongs to only one group. On the other hand, soft clustering method such as fuzzy clustering is appropriate for overlapping clustering task, so data can belong to all clusters with a certain value of membership and it can give descriptions of objects in clusters in more detail. Fuzzy clustering is appropriate for data with complex structures or when there are vague or overlapping class boundaries. Moreover, fuzzy clustering can be more robust to outliers and noise in data. Choosing an appropriate clustering algorithm solely depends on the data type to be clustered and the purpose of the clustering applications.^15^

 Some data sets cannot be adequately split into some non-overlapping clusters, while partitions may overlap with each other to some degree, and some data points contribute to more than one cluster. Fuzzy clustering algorithms are helpful for datasets with subgroupings of points having indistinct boundaries and overlap between the clusters such as human behavior data. Human behavior (e.g., traffic behavior) is an inherently complex subject; so, it cannot be clearly clustered into completely separate clusters, and there will be overlap between the clusters so that a person’s behavior can potentially belong to multiple clusters of behavior. For these cases, we can use fuzzy clustering as a membership value-based clustering method which allows an object to be a member of more than one cluster but with different membership degrees.^15^

 As we stated, it is crucial to use machine learning methods such as clustering method to identify traffic behavior patterns and investigate their effective factors. Hence, this study aimed to identify the hidden pattern of pedestrians’ behaviors in Urmia, Iran, by using fuzzy cluster analysis and assess the factors affecting this pattern.

## Methods

###  Participants

 This study is a secondary study based on the data collected by Bakhtari et al (preprint).^16^ This descriptive cross-sectional study was carried out among participants aged 18 years and above (N = 600) living in Urmia, Iran, from May to October 2018. In this study, the cluster sampling method was applied for sampling, so the health centers were considered clusters, and some of them were randomly selected. Then, from each selected center, depending on the population covered, the participants were randomly selected in terms of the inclusion and exclusion criteria.

 The inclusion criteria were being 18 years old or above, willing to participate in the study, and being capable of standing and walking. The exclusion criteria were having a history of severe mental illness, depression, Alzheimer’s, dementia, restrictive musculoskeletal disorders, neurological deficits (stroke), Parkinson’s and paralytic disease, acute myocardial infarction, uncontrolled hypertension, and severe hearing and visual impairment. These exclusion criteria were checked by their self-reported medical records.

 Selected subjects that were willing to participate in the study, were invited to visit the health centers. Based on a previous study^16^ and considering the standard deviation of pedestrian behaviors, cluster sampling design effect, as well as the rate of incomplete questionnaires, they estimated the sample size at 600.

###  Data collection and questionnaire

 Some information including demographic characteristics (age, gender, marital status, and education), pedestrian traffic behavior, walking minutes/day, and transportation mode were gathered by relevant questioners.

 Pedestrian traffic behavior data were collected using the “Pedestrians Behavior Questionnaire” (PBQ) which is a valid and reliable instrument.^17^ PBQ includes 29 items measuring traffic behavior with a five-point Likert scale from 1 to 5 (1 = never, 2 = rarely, 3 = sometimes, 4 = often, and 5 = always). It represents 5 domains of traffic behavior: (1) adherence to traffic rules (7 items) (e.g., I cross the street after the vehicles are fully stopped), (2) traffic violations (10 items, reverse scored) (e.g., I don’t use the pedestrian bridge because most people don’t), (3) positive behaviors (2 items) (e.g., I let the vehicle pass even if I have the priority right), (4) traffic distraction (4 items, reverse scored) (e.g., I use my mobile phone while crossing the street), and (5) aggressive behaviors (2 items, reverse scored) (e.g., If I get angry at the behavior of a driver, I would kick or punch the car). The score of each domain was calculated as the mean score of its items, and the mean scores of all 29 items showed the total score of PBQ. Hence, the score of each domain of PBQ and the total score of PBQ ranged from 1 to 5. A higher score in all domains and total score indicated better traffic behavior and vice versa.

###  Statistical analyses 

 Statistical analyses were performed using R software version 4.2.2 (packages: fclust^18^). Quantitative and qualitative variables were presented as mean and standard deviation (SD) as well as number and percentage, respectively. This study used fuzzy c-means (FCM) clustering for clustering pedestrians’ behavior. The silhouette index, partition entropy, partition coefficient (PC), and modified partition coefficient (MPC) were used to select the optimal number of clusters.^19-21^ Furthermore, the chi-square test and multiple logistic regression were used to examine the factors related to the obtained clusters.

###  Clustering task 

 Cluster analysis tries to separate data into groups or clusters such that both the homogeneity within the clusters and the heterogeneity between clusters are maximized.^22^ This technique is an unsupervised machine learning algorithm because it uses machine learning algorithms to cluster unlabeled data and discover unknown patterns.^23,24^ Clustering methods are distinguished regarding how they allocate data to clusters and are divided into two categories: soft clustering (overlapping clustering) and hard clustering (exclusive clustering). In classical or hard cluster analysis, each data must be allocated to exactly one cluster while soft cluster analysis techniques allow overlapping clusters.^25^

 K-means clustering belongs to the hard clustering, and FCM clustering belongs to the soft clustering category. In k-mean cluster analysis, each data point belongs to only one cluster with membership values of zero or one, while in fuzzy cluster analysis, the membership value of assigning a data point to clusters is between [0, 1], so data can belong to more than one cluster simultaneously with certain membership values, and it can give descriptions of objects in clusters in more detail.^15,26-29^

 Hard clustering approaches such as k-means are simple, easy to modify, and less complex to interpret, but they are sensitive to the centroid initialization and outlier,^30,31^ and FCM is more flexible than conventional k-means. Although soft clustering such as FCM is supposedly slower and computation time increases more rapidly for FCM than for the k-means algorithm with the growing number of clusters and sample size, this should not be of concern with the power of today’s computers.^32^

###  K-means clustering algorithm

 The k-means algorithm is an iterative algorithm that tries to partition the dataset into the fixed number (k) of distinct non-overlapping clusters in a dataset where each data point belongs to only one cluster. This algorithm attempts to make the intra-cluster data points as similar as possible while also maintaining the clusters as different as possible. It allocates data points to a cluster such that the sum of the squared distance between the data points and the cluster’s centroid is the minimum. The less variation within clusters, the more homogeneous the data points are within the same cluster.^23,24^

###  Fuzzy C-means clustering algorithm

 The FCM is the weighted sum of squared errors within clusters, which is defined as follows:


(1) $$J_{m}\left(U,V;X\right)=\sum_{k=1}^{n}\sum_{i=1}^{c}u_{ik}^{m}|\left|x_{k}-v_{i}\right|{|}_{A}^{2},\;1<m<\infty \;,$$

where *V* = (*v*_1_*, v*_2_, …, *v*_c_) is a vector of the unknown cluster (centers)*v*_i_ ∈ R^p^. The value of *u*_ik_ represents the degree of membership of the data point *x*_k_ of set *X* = {*x*_1_*,x*_2_*,…, x*_n_} to the *i*th cluster.

 Ameasure of similarity between a data point and thecluster prototypes as theinner product was defined by a norm matrix *A*. A fuzzy *c*-partitionof *X *^33^ is suitably represented by a matrix *U* = [*u*_ik_]. That if $|\left|x_{k}-v_{i}\right|{|}_{A}\rangle 0$ for all *i*and *k*, then (*U*,*V) *may minimize *J*_m_, when *m* > 1 and


(2) $$v_{i}=\frac{\sum_{k=1}^{n}{\left(u_{ik}\right)}^{m}x_{k}}{\sum_{k=1}^{n}{\left(u_{ik}\right)}^{m}}for\;1\leq i\leq c\;,$$

(3) $$u_{ik}=\frac{1}{\sum_{j\;1}^{c}{\left(\frac{|\left|x_{k}-v_{i}\right|{|}_{A}^{2}}{|\left|x_{k}-v_{j}\right|{|}_{A}^{2}}\right)}^{\frac{1}{m\;1}}}for\;1\leq i\leq c,\leq k\leq n.$$

The Picard iteration approach minimizes *J*_m_by initializing the matrix ***U *** randomly and computing the (Eq2) and(Eq3) after each iteration. The Picard iteration method is an iterative algorithm used in FCM clustering to update the membership values and cluster centers. It is an extension of the classic c-means clustering algorithm that allows for fuzzy membership values, indicating the degree of belongingness of each data point to each cluster.When iteration reaches a stable condition, it is terminated; that is, when the changes in the cluster centers or the membership values at two successive iterations are smaller than a predefined threshold value.

 The FCM algorithm always converges to a minimum point. A different initial guess of *u*_ij_ may lead to a different minimum. Finally, to allocate each data point to a particular cluster, defuzzification is necessary, and this can be done by assigning a data point to a cluster for whh the value of the membership is maximal. Defuzzification is the process of converting fuzzy membership values into crisp values in FCM.^15,26-29^

###  Clustering validity indices

 Clustering validity indices are employed for the quality evaluation of partitions produced by clustering algorithms as well as for determining the number of clusters in the dataset.^20,34-37^ They are calculated for various numbers of clusters, and the optimal number of clusters is determined by comparing the values of an index for all possible numbers of clusters. The following section outlines a brief explanation of the more commonly used fuzzy clustering validity indices that are used to measure the clustering performance and determine the optimal number of clusters.

###  Partition coefficient

 In fuzzy clustering, a PC that was initially designed by Bezdek^20^ is defined as:


$$PC=\frac{1}{N}\sum_{i=1}^{c}\sum_{j=1}^{N}u_{ij}^{2}$$

The PC values range in [1/c, 1]. The closer the index value is to 1, the clearer the clustering, and 1/c indicates that there is no clustering tendency in the considered dataset.

###  Modified partition coefficient

 A PC correction (MPC)^38^ is defined using a linear transformation to remove the dependence of PC on c. The modified PC is expressed as:


$$MPC=\frac{c}{c-1}PC-\frac{1}{c-1}$$

The range of MPC is [0, 1] where MPC = 0 corresponds to maximum fuzziness and MPC = 1 to a hard partition.

###  Partition entropy coefficient

 The partition entropy (PE) coefficient^19^ is defined as:


$$PE=-\frac{1}{N}\sum_{i=1}^{c}\sum_{j=1}^{N}u_{ij}\log u_{ij}$$

The PE index values range in [0, log c]. The closer the value of PE to 0, the clearer the clustering is. The index value close to the upper bound (i.e., log c) indicates the lack of any clustering structure in the data sets.

###  Silhouette index

 The Silhouette coefficient combines the factors of intra-cluster polymerization and inter-cluster resolution to measure the clustering effect.^21^ The Silhouette index gets the optimal clustering number by computing the difference between the average distance within the cluster and the minimum distance between the clusters; that is, the optimal clustering effect, which is defined as:


$$\bar{S}=\frac{1}{n}\sum_{i=1}^{n}\left(\frac{b\left(i\right)-a\left(i\right)}{\max\left\{a\left(i\right),b\left(i\right)\right\}}\right)$$

where a (i) represents the average distance of sample i to other samples in the cluster, and b (i) represents the minimum distance of the sample from the sample i to the other clusters.

## Results

Table 1 depicts a summary of the data, including detailed information and descriptions of the traffic behavior of the participants, as well as the frequency and percentage of their demographic characteristics. Demographic characteristics are age, gender, marital status, education, transportation characteristics, and pedestrian traffic behavior variables are adherence to traffic rules, traffic violation, positive behavior, traffic distraction, aggressive behavior, and total score of PBQ.

**Table 1:** Descriptive information of pedestrians’ behavior and their demographic characteristics

**Quantitative variables **	**Mean**	**SD**
Adherence to traffic rules	3.48	0.73
Traffic violation	3.93	0.68
Positive behavior	3.61	0.74
Traffic distraction	4.04	0.79
Aggressive behavior	4.32	0.95
Total Score PBQ	3.80	0.51
**Qualitative variables **	**Number**	**Percent**
Gender		
Male	239	39.8
Female	361	60.2
Marital status		
Single	155	25.8
Married	423	70.5
Other	22	3.7
Education status		
Illiterate	11	1.8
1-6 grades	91	15.2
7-12 grade diploma	214	35.7
Associate	66	11.0
Bachelor	150	25.0
Master	49	8.2
Doctoral and higher	19	3.2
Walking (min)		
< 30	263	43.8
30-60	199	33.2
60-120	92	15.3
120	46	7.7
Transportation		
Personal car	392	65.3
Taxi	84	14.0
Large vehicle	51	8.5
Motorcycles and bicycles	12	2.0
Walking	61	10.2
Age (y)		
18-25	123	20.5
26-33	185	30.8
34-41	153	25.5
≥ 42	139	23.2

###  The optimal number of clusters

 Pedestrian behavior was clustered based on variables such as adherence to traffic rules, traffic violation, positive behavior, traffic distraction, aggressive behavior, and total PBQ score. To select the optimal number of clusters (c), different numbers of clusters were tested from two to six. The lower and upper limit of the number of clusters was determined according to the previous studies and validation indexes. The fuzzy silhouette index, PE, PC, and modified PC were used to select the optimal number of clusters, which is presented in Table 2. For c = 2, the fuzzy silhouette index, PC, and modified PC were larger than other cluster numbers (a higher score is better), and for c = 2, PE was lower (a lower score is better). As a result, the optimal number of clusters was determined “2”.

**Table 2:** The value of cluster validity indexes for choosing the optimal number of clusters

**Cluster Number (c)**^a^	**FSI**	**PE**	**PC**	**MPC**
2	0.639772	0.477040	0.691147	0.382293
3	0.485307	0.850437	0.493321	0.239982
4	0.408518	1.096336	0.400192	0.200256
5	0.436820	1.295305	0.340591	0.175739
6	0.423527	1.465443	0.294850	0.153819

*Note.* FSI: Fuzzy silhouette index; PE: Partition entropy; PC: Modified partition; MPC: Modified partition coefficient. ^a^for c = 2, the FSI, PC, and MPC are larger than other cluster numbers (a higher score is better), and PE is lower (a lower score is better).

###  Clustering description

Table 3 illustrates the traffic behavior of the participants according to revealed clusters and statistical differences between them. As shown, the first cluster (C1) had lower scores in adherence to traffic rules, violation, positive behavior, distraction, aggressive behavior, and total PBQ compared to the second cluster; therefore, we can name it the higher-risk traffic behavior cluster. Of the investigated participants, 214 (35.66%) of them belonged to this cluster.

 The second cluster (C2) had higher traffic behavior scores for total PBQ and its domains compared to the first cluster, so we can regard this cluster as the lower-risk traffic behavior cluster. Most of the participants belonged to this cluster (64.33%).

**Table 3:** Mean and SD of traffic behavior in each cluster

**Fuzzy c-means**	**Cluster 1=214 (Higher risk)**		**Cluster 2=386 (Lower risk)**		***P***** value**
	**Mean**	**SD**	**Mean**	**SD**	
Adherence to traffic rules	3.12	0.70	3.68	0.67	0.001
Traffic violation	3.34	0.61	4.26	0.46	0.001
Positive behavior	3.10	0.68	3.89	0.60	0.001
Traffic distraction	3.40	0.76	4.40	0.53	0.001
Aggressive behavior	3.36	0.98	4.86	0.31	0.001
Total score PBQ	3.25	0.30	4.10	0.29	0.001

*Note.* SD: standard deviation; PBQ: Pedestrian behavior questionnaire.

 The mean ± SD of the total PBQ score of the pedestrian in the lower-risk cluster was 4.10 ± 0.29, while it was 3.25 ± 0.30 in the higher-risk cluster (*P* < 0.001). Moreover, all domains of PBQ significantly differed in the two clusters (*P* < 0.001), as depicted in Table 3.

 According to the results of the chi-square test, there were significant differences between lower-risk and higher-risk clusters according to the pedestrians’ age, education, gender, marriage status, and kind of vehicle used for transportation which indicates a relationship between these factors and pedestrians’ traffic behavior pattern; therefore, the proportion of female pedestrians in lower-risk cluster was higher than that in higher-risk cluster (65.3% vs. 50.9%). Married pedestrians were more prevalent in the lower-risk cluster (76.4% vs. 59.8%), and more than 71.2% of the pedestrians in the lower-risk cluster and 54.7% in the higher-risk cluster used personal cars. Moreover, more than 54.6% of the participants in the lower-risk cluster were over 34 years old, while this rate was 37.9% in the higher-risk cluster. About 51.1% of lower-risk participants vs. 38.8% of higher-risk pedestrians had academic education which indicates their safer traffic behavior (Table 4).

**Table 4:** Distribution of underling variables according to the clusters

**Variables**	**Cluster 1=Higher-risk**		**Cluster 2=Lower-risk**		***P***** value**
	**Number**	**Percent**	**Number**	**Percent**	
Gender					0.001
Male	105	49.1	134	34.7	
Female	109	50.9	252	65.3	
Age (y)					0.001
18-25	55	25.7	68	17.6	
26-33	78	36.4	107	27.7	
34-41	44	20.6	109	28.2	
> 42	37	17.3	102	26.4	
Marital status					0.001
Single	72	33.6	83	21.5	
Married	128	59.8	295	76.4	
Other	14	6.5	8	2.1	
Educational status					0.044
Illiterate	5	2.3	6	1.6	
1-6 grades	45	21.0	46	11.9	
7-12 grade diploma	77	36.0	137	35.5	
Associate	24	11.2	42	10.9	
Bachelor	44	20.6	106	27.5	
Master	15	7.0	34	8.8	
Doctoral and higher	4	1.9	15	3.9	
Transportation					0.001
Personal car	117	54.7	275	71.2	
Taxi	43	20.1	41	10.6	
Large vehicle	22	10.3	29	7.5	
Motorcycles and bicycles	9	4.2	3	0.8	
Walking	23	10.7	38	9.8	
Walking (min)					0.080
< 30	86	40.2	177	45.9	
30-60	66	30.8	133	34.5	
60-120	42	19.6	50	13.0	
> 120	20	9.3	26	6.7	

*Note.* SD: Standard deviation.

 Results of multiple logistic regression to assess independent predictors of having higher-risk traffic behavior demonstrated the significant effects of age, gender, marital status, type of transportation in the city, and education on being in the higher-risk cluster of traffic behavior (compared to the lower-risk cluster).Hence, subjects ≤ 33 years old compared to > 33 years old were more likely to have higher-risk traffic behavior (Odds ratio [OR] = 1.92, 95% confidence interval [CI]: 1.33-2.75, *P* < 0.001). Subjects with primary education or less compared to secondary or higher-educated pedestrians were more likely to be in the higher-risk cluster (OR = 1.74, 95% CI: 1.10- 2.74, *P*= 0.010). Furthermore, male pedestrians had higher odds of more risky traffic behavior compared to females (OR = 1.90, 95% CI: 1.31-2.75, *P*= 0.001). In addition, unmarried pedestrians compared to married people (OR = 3.61, 95% CI: 1.40- 9.23, *P*= 0.007) and users of public transportation compared to users of personal cars (OR = 2.01, 95% CI: 1.30-3.08, *P*= 0.002) were more likely to be in higher-risk traffic behavior cluster (Table 5).

**Table 5:** Multiple logistic regression to assess independent predictors of having higher-risk traffic behavior

**Variables**	**B**	**SE**	***P***** value**	**OR (95% CI)**
Gender				
Male	0.64	0.18	0.001	1.90 (1.31, 2.75)
Female				1.00
Age (y)				
≤ 33	0.65	0.18	0.001	1.91 (1.33, 2.75)
> 33				1.00
Marital status				
Unmarried	1.28	0.48	0.007	3.60 (1.40, 9.23)
Married				1.00
Educational status				
Illiterate or 1-6 grades	0.55	0.23	0.017	1.74 (1.10, 2.74)
> 7 grades				1.00
Transportation				
Public transportation	0.69	0.21	0.002	2.01 (1.30, 3.07)
Walking or biking	0.42	0.28	0.142	1.52 (0.86, 2.68)
Personal car				1.00
Walking (min)				
< 30	0.11	0.36	0.754	1.12 (0.54, 2.28)
30-60	-0.04	0.36	0.904	0.95 (0.47, 1.94)
60-120	0.27	0.39	0.485	1.31 (0.60, 2.85)
> 120				1.00

*Note*. SE: Standard error, OR: Odds ratio; CI: Confidence interval.

## Discussion

 Pedestrian behavior plays an important role in pedestrian safety. In this study, we first clustered the behaviors of pedestrians based on PBQ domains using the fuzzy clustering method. According to the validation indices, the optimum number of clusters was 2. Cluster analysis with two clusters revealed two behavioral patterns; that is, pedestrians in the first cluster had a lower score of PBQ, and their traffic behavior was riskier, while pedestrians’ behaviors in the second cluster were safer, and they obtained a higher score of PBQ and its domains. Afterward, we assessed the association between underlying factors (e.g., demographic characteristics, type of transportation, and the like) and unsafe behavior using multiple logistic regression. The results demonstrated the significant effect of age, gender, marital status, type of transportation in the city, and education on being in the higher-risk cluster of traffic behavior. Clustering our pedestrians’ behavior dataset into two homogeneous subsets helped to identify associated factors that are not easily detectable when using the dataset as a whole.

 As demonstrated in this study, clustering techniques can be used not only for descriptive analysis but also as a prepossessing segmentation tool for a more detailed standard statistical analysis.^6^ Clustered data rather than the raw dataset can provide clear and more meaningful information.^8,39-42^

 The application of FCM clusters is not limited to the traffic behavior of pedestrians. We can apply it to all types of traffic data to find better solutions for improving traffic safety. Due to the complex nature of traffic data such as high dimensionality, spatial-temporal structure, and overlapping clusters, cluster fuzzy clustering has been applied frequently.^43-46^ Furthermore, researchers have used k-means clustering algorithms to identify homogeneous coincidence clusters.^42,47,48^ In a study,^41^ Latent class cluster and multinomial logit models were used to investigate the statistical relationship between pedestrian injury severity outcome and contributing factors (e.g., pedestrian behavior, demographics, accident characteristics, and the built environment). According to the obtained results, there is a relationship between severe accidents and variables such as using alcohol or drugs, age over 65, and adverse weather conditions.

 Depaire et al^14^ succeeded in investigating the performance of latent class clustering for traffic accident segmentation. The clusters obtained from the types of traffic accidents were sensible and could examine the effect of variables such as the age and type of road on traffic accidents.

 However, regarding the pattern of pedestrian behavior, to the best of our knowledge, this study is the first one that used cluster analysis to identify patterns of pedestrians in terms of their traffic behavior. For analyzing pedestrian behavior, other statistical methods such as binary logit, ordered logit or probit, mixed logit, and multinomial logit models have been used.^9,49-53^ Hence, we did not find any similar study to compare our results with them due to the different nature of the results.

 Regarding revealed clusters, according to the scores of PBQ and its domains in two clusters, the clusters were named lower-risk and higher-risk pedestrians, and about 35% of pedestrians belonged to the higher-risk cluster. According to the range of scores of the questionnaire and its dimensions, which are between 1 to 5, the pedestrians in both clusters had scored higher than the middle score (score 3) and had acceptable traffic behavior in comparison with each other. Therefore, we can conclude that there are two clusters of pedestrians in Urmia: one with safe and cautious traffic behavior (total PBQ score of 4.1) and the other with moderate traffic behavior (total PBQ score of 3.25).

 This finding can help develop educational and intervention programs for pedestrians as we encounter two groups of the population with moderate and good traffic behavior, so planning and policymaking should be performed considering these two groups.

 Regarding factors affecting these traffic behavior patterns, the current results indicated that age, gender, marital status, type of transportation in the city, and education are related to the pattern of traffic behavior. In this study, female pedestrians had safer traffic behavior than males, and this finding is consistent with previous studies, which show males have more risky traffic behavior.^54-57^

 Furthermore, according to our results, age was a significant variable related to pedestrian traffic behavior. Hence, younger pedestrians were more likely to be in the higher-risk cluster of traffic behavior. Consistent with our findings, some studies^58-60^ have shown that young pedestrians are more distracted than older people and show aggressive behavior. This behavior may be due to the risk-taker nature of this age group or the use of cell phones.

 Moreover, based on the findings of our study, education was associated with the traffic behavior of pedestrians; that is, higher-educated pedestrians had safer traffic behavior. In this regard, we can declare that the increase in the level of education makes their behaviors and decisions more reasonable, especially in adherence to traffic rules and aggressive behaviors, and this result is consistent with other studies.^61^

 In our data, married pedestrians had safer behavior. It can be influenced by age-related changes, or having a family may make people more responsible and cautious. This result is qualitatively consistent with similar studies with increased risks of driver injury among never-married people.^62^

 Results of previous investigations regarding the role of marriage in pedestrians’ traffic behavior are consistent with the current study. In line with our findings, Ghahramani et al revealed that married people are better than single ones in terms of traffic behavior.^63^

 Regarding the kind of transportation, our results indicated that transportation with personal car decreases the odds of being in the higher-risk traffic behavior cluster. Although we did not find any relevant studies, due to the experience of driving and having a better perception of crashes, the people with individual cars avoid risky behavior. The use of the self-reporting method for data collection was the major limitation of this study because it may lead to bias in reporting traffic behavior.

**Highlights**

- It is crucial to identify the pattern of traffic behaviors of pedestrians to enhance management efficiency.
- We evaluated the pedestrians’ traffic behavior pattern with the fuzzy c-means algorithm.
- Two clusters, consisting of lower-risk and higher-risk behaviors, were revealed. The majority of pedestrians (64.33%) were in the lower-risk cluster.
- Age, gender, marriage, education, and kind of transportation were associated with traffic behaviors of pedestrians.
- These findings can help promote safety measures and train pedestrians.

## Conclusion

 We identified traffic behavior patterns of Urmia pedestrians consisting of lower-risk and higher-risk behaviors with FCM. Understanding which group of pedestrians have more unsafe behaviors and what causes them may help planners and policymakers think of better training solutions for them. The current study showed that using statistical methods, including clustering, can provide us with more details in addition to statistical descriptions. The findings from this study would help promote safety measures and training pedestrians.

## Acknowledgements

 We would like to acknowledge all participants who took part in this study. Moreover, we thank the Vice-Chancellor for Research and Technology of Tabriz University of Medical Sciences for financial support of this study (Grant number 65780).

## Authors’ Contribution

**Conceptualization:** Parvin Sarbakhsh.

**Data curation:** Fatemeh Bakhtari Aghdam.

**Formal analysis:** Parisa Saeipour.

**Funding acquisition:** Parvin Sarbakhsh.

**Investigation:** Parvin Sarbakhsh, Parisa Saeipour.

**Methodology:** Parvin Sarbakhsh, Parisa Saeipour.

**Project administration:** Parvin Sarbakhsh.

**Software:** Parisa Saeipour.

**Supervision:** Parvin Sarbakhsh, Fatemeh Bakhtari Aghdam.

**Writing–original draft:** Parisa Saeipour, Saman Salemi.

**Writing–review & editing:** Parisa Saeipour, Saman Salemi, Parvin Sarbakhsh.

## Competing Interests

 The authors declare that they have no competing interests.

## Ethical Approval

 This study was conducted in accordance with the ethical standards of the Helsinki Declaration. All participants completed the informed consent form. Ethical approval for the current secondary study was provided by the Ethical Committee of Tabriz University of Medical Sciences (Ethics No: IR.TBZMED.REC.1399.623). The Ethical code of the primary study was IR.TBZMED.REC.1397.969 provided by the Ethical Committee of Tabriz University of Medical Sciences.

## Funding

 This project has been implemented with financial support from the Vice-Chancellor for Research and Technology of Tabriz University of Medical Sciences with grant number 65780.
